# The DNA Sensor AIM2 Protects against Streptozotocin-Induced Type 1 Diabetes by Regulating Intestinal Homeostasis via the IL-18 Pathway

**DOI:** 10.3390/cells9040959

**Published:** 2020-04-14

**Authors:** Jefferson Antônio Leite, Gabriela Pessenda, Isabel C. Guerra-Gomes, Alynne Karen Mendonça de Santana, Camila André Pereira, Frederico Ribeiro Campos Costa, Simone G. Ramos, Dario Simões Zamboni, Ana Maria Caetano Faria, Danilo Candido de Almeida, Niels Olsen Saraiva Câmara, Rita C. Tostes, João Santana Silva, Daniela Carlos

**Affiliations:** 1 Department of Biochemistry and Immunology, Ribeirão Preto Medical School, USP—Avenida Bandeirantes 3900, Monte Alegre, Ribeirão Preto, São Paulo 14049-900, Brazil; jeffersonleite@usp.br (J.A.L.); gapessenda@usp.br (G.P.); Isabelguerragomes@gmail.com (I.C.G.-G.); lynne_karen@hotmail.com (A.K.M.d.S.); fredericoribeiro@usp.br (F.R.C.C.); niels@icb.usp.br (N.O.S.C.); 2 Institute of Biomedical Science IV—University of São Paulo (USP), São Paulo, São Paulo 14049-900, Brazil; 3 Department of Pharmacology, Ribeirão Preto Medical School, University of São Paulo (USP), Ribeirão Preto, São Paulo 14049-900, Brazil; mila_cap@yahoo.com.br (C.A.P.); rtostes@usp.br (R.C.T.); 4 Department of Pathology and Legal Medicine, Ribeirão Preto Medical School, University of São Paulo (USP), Ribeirão Preto, São Paulo 14049-900, Brazil; sgramos@fmrp.usp.br; 5 Department of Molecular and Cell Biology, Ribeirão Preto Medical School, University of São Paulo (USP), Ribeirão Preto, São Paulo 14049-900, Brazil; dszamboni@fmrp.usp.br; 6 Department of Biochemistry and Immunology, Institute of Biological Science—Federal University of Minas Gerais (UFMG), Belo Horizonte, Minas Gerais 31270-901, Brazil; anacaetanofaria@gmail.com; 7 Department of Immunology—Federal University of São Paulo (UNIFESP), São Paulo, São Paulo 04021-001, Brazil; gudaalmeida@gmail.com; 8 Fiocruz- Bi-Institutional Translational Medicine Platform, Ribeirão Preto, São Paulo 14049-900, Brazil

**Keywords:** Innate immunity, AIM2 receptor, Type 1 diabetes

## Abstract

Pattern recognition receptors (PRRs), such as Nod2, Nlrp3, Tlr2, Trl4, and Tlr9, are directly involved in type 1 diabetes (T1D) susceptibility. However, the role of the cytosolic DNA sensor, AIM2, in T1D pathogenesis is still unknown. Here, we demonstrate that C57BL/6 mice lacking AIM2 (AIM2^−/−^) are prone to streptozotocin (STZ)-induced T1D, compared to WT C57BL/6 mice. The AIM2^−/−^ mice phenotype is associated with a greater proinflammatory response in pancreatic tissues, alterations in gut microbiota and bacterial translocation to pancreatic lymph nodes (PLNs). These alterations are related to an increased intestinal permeability mediated by tight-junction disruption. Notably, AIM2^−/−^ mice treated with broad-spectrum antibiotics (ABX) are protected from STZ-induced T1D and display a lower pancreatic proinflammatory response. Mechanistically, the AIM2 inflammasome is activated in vivo, leading to an IL-18 release in the ileum at 15 days after an STZ injection. IL-18 favors RegIIIγ production, thus mitigating gut microbiota alterations and reinforcing the intestinal barrier function. Together, our findings show a regulatory role of AIM2, mediated by IL-18, in shaping gut microbiota and reducing bacterial translocation and proinflammatory response against insulin-producing β cells, which ultimately results in protection against T1D onset in an STZ-induced diabetes model.

## 1. Introduction

Type 1 diabetes (T1D) is an autoimmune disease that occurs when immunological tolerance to self-tissues fails, resulting in the destruction of insulin-producing β cells in genetically predisposed individuals [1]. While genetic factors seem to play a role in T1D, solid evidence supports a critical involvement of environmental factors in the disease pathogenesis [2]. T1D triggers include viral infections, gut microbiota alterations and diet [3]. These environmental factors may act in the beginning or progression of the autoimmune disease that results in β cells damage [4]. Many experimental models have been used to study T1D, including mouse models with diabetes induced by chemicals, such as streptozotocin (STZ), cyclophosphamide, and alloxan, and nonobese diabetic (NOD) mice, in which the disease develops spontaneously [5]. STZ-induced diabetes represents an immune-mediated mouse model of T1D. In this context, splenocytes from STZ-injected mice causes insulin resistance and diabetes upon adoptive transfer [6,7]. In addition, anti-insulin antibodies were found in the sera of naive C57BL/6 mice, which are susceptible to STZ-induced diabetes, reinforcing an autoimmune response in this model [8].

The gut microbiota is considered an important component in T1D pathogenesis [9,10,11]. However, the mechanisms by which gut microbiota alterations contribute to disease development are still unclear. In this context, previous studies in murine models have shown that T1D is aggravated by the administration of antibiotics [12]. On the other hand, the depletion of the gut microbiota by antibiotic treatment prevents the disease onset [13]. Additionally, studies on T1D patients have reported alterations in the gut microbiota composition [14], with T1D patients exhibiting a disruption of the intestinal barrier, called a “leak-gut” [15]. These reports suggested a potential link/association between the gut microbiota and T1D development. However, the exact mechanisms by which the gut microbiota contributes to the autoimmune process against insulin-producing β-cells remains unclear.

The adaptive immune system plays a crucial role in T1D development, particularly through diabetogenic CD4^+^ and CD8^+^ T cells that damage insulin-producing β cell through several mechanisms [16]. On the other hand, the role of the innate immune system in T1D onset remains poorly understood and controversial. It is known that microbe-associated molecular patterns (MAMPs) derived from microbiota activate the host’s immune system via innate immune receptors, such as TLRs (toll-like receptors) and NLRs (nucleotide-binding domain and leucine-rich repeat-containing receptors). TLRs and NLRs are expressed in epithelial and immune cells (macrophages and dendritic cells), which are present in the lamina propria (LP) of the intestinal mucosa [17]. Importantly, several studies have demonstrated the role of these innate immune receptors in T1D susceptibility. In the NOD mouse model, the deficiency of TLR2, TLR4, and Myd88 protects against insulitis, and these mice have a lower incidence of T1D when maintained under germ-free conditions [18]. Similarly, TIR domain-containing adapter-inducing interferon-β (TRIF) deficiency in NOD mice protects against T1D development through the control of gut microbiota dysbiosis and immune cell activation [19]. In addition, TLR9 activation and the release of interferon-beta (IFN-β) promote the expansion of diabetogenic CD8^+^ T cells in the pancreatic lymph nodes (PLNs) of NOD mice, thus increasing T1D severity [20].

We have recently shown that NOD2 and NLRP3 activation contributes to the generation of a proinflammatory environment favoring T1D development [21,22]. NOD2^−/−^ mice are protected from T1D induced by multiple low doses of streptozotocin (MLD-STZ). These mice have less diabetogenic T cells in the PLNs and decreased levels of proinflammatory cytokines, such as IL-12, IL-23, IFN-γ, IL-1β and IL-17, in the pancreatic tissue. Mechanistically, NOD2 activation in STZ-induced T1D depends on the microbiota. Gut microbiota translocation to the PLNs activates NOD2 in myeloid cells [dendritic cells (DCs) and macrophages], generating a proinflammatory environment that results in insulin-producing β cell damage and T1D onset [21]. We also demonstrated that NLRP3 inflammasome activation by mitochondrial DNA promotes IL-1β and IL-18 release, contributing to the generation of pathogenic Th17/Th1 cells in the PLNs and increasing T1D susceptibility in STZ-induced diabetes [22]. Accordingly, an association study in Brazil identified two single-nucleotide polymorphisms in NLRP3 that are associated with T1D in humans [23].

Another innate immune receptor that, upon its activation, results in inflammasome assembly, but which does not belong to the NLRs family, is the DNA sensor, AIM2 (*absent in melanoma 2*) [24,25]. The activation of AIM2 has been reported to be involved in autoimmune and inflammatory diseases [26]. In patients with systemic lupus erythematous (SLE), the expression of AIM2 is increased and is correlated with the severity of the disease. Additionally, a deficiency of AIM2 in mice significantly attenuates apoptotic DNA-induced macrophage activation. In agreement, the blockade of AIM2 notably ameliorates SLE syndrome by reducing macrophage activation and dampening the inflammatory response in mice with apoptotic DNA-induced lupus [27]. In psoriatic patients, the expression of AIM2 and IL-1β is increased in keratinocytes from the skin lesion, and the activation of AIM2 in cultured keratinocytes leads to IL-1β release, suggesting that AIM2 is involved in the psoriasis pathogenesis [28]. In colitis, AIM2 senses microbial DNA and activates the inflammasome producing IL-1β and IL-18, which contributes to the production of antimicrobial peptide by intestinal epithelial cells, thus regulating gut microbiota dysbiosis and controlling the development of dextran sulfate sodium (DSS)-induced colitis [29,30]. Despite these results, which demonstrate that the AIM2 receptor is an important immunological target in autoimmune diseases, there is no direct or indirect evidence of AIM2 involvement in T1D.

Here, we demonstrate that STZ-induced T1D increases the AIM2 gene and protein expression and caspase-1 activation in pancreatic and intestinal tissues (ileum). Interestingly, AIM2 activation increases IL-18 production in intestinal mucosa, regulating claudin-2 and RegIIIγ expression to restrain gut microbiota translocation to PLNs. In turn, this intestinal homeostasis minimizes the generation of diabetogenic Th1/Tc1 lymphocyte responses against pancreatic insulin-producing β cells, leading to protection against T1D development.

## 2. Methods

### 2.1. Animals

Male C57BL/6, AIM2^−/−^ and IL-18^−/−^ mice (8–12 weeks old) were obtained from the Isogenic Breeding Unit at Ribeirao Preto Medical School, University of São Paulo, Ribeirao Preto, Brazil. All mice were housed in standard cages and had free access to water and food. AIM2^−/−^ and IL-18^−/−^ mice were bought from The Jackson Laboratory, under the line names, B6.129P2-Aim2Gt(CSG445)Byg/J and B6.129P2-Il18tm1Aki/J, respectively. The present study was carried out in accordance with the principles of the Basel Declaration and recommendations of the National Animal Experimentation Control Council (CEUA) at the Laboratory of Immunoregulation of Metabolic Disorders in the Department of Biochemistry and Immunology, Ribeirão Preto Medical School, University of São Paulo, São Paulo, Brazil. This research project was approved by the Animal Research Ethics Committee of the Ribeirao Preto Medical School, University of São Paulo (no. 215/2016). For euthanasia, a supra dose of anesthesia consisting of Ketamine-Xylazine (Agribrands do Brazil, São Paulo, Brazil) was intraperitonially injected in the mice.

### 2.2. Induction of Diabetes by Multiple Low Doses of STZ (MLD-STZ)

Mice were given daily intraperitoneal (i.p.) injections of 40 mg/kg of streptozotocin (Sigma-Aldrich, San Luis, MO, USA) dissolved in a 0.1 M sodium citrate buffer (pH 4.5) for five consecutive days. Control mice only received a sodium citrate buffer (vehicle). The blood glucose levels and diabetes incidence were monitored weekly. Mice were defined as diabetic when their glucose levels were ≥200 mg/dL, after two consecutive determinations under non-fasting conditions.

### 2.3. Flow Cytometry Analysis of Intracellular and Extracellular Markers

Flow cytometry analysis was performed by the isolation of pancreatic lymph node cells (PLNs). PLNs were harvested, and cells were dissociated using a cell strainer and washed in phosphate-buffered saline (PBS) once and counted in a hemocytometer. Next, 2 × 10^6^ cells were resuspended in 100 µL of PBS and incubated with 5% normal rabbit serum for 30 min to block non-specific binding. Cells were fixed in PBS containing 4% paraformaldehyde and permeabilized in PBS containing 1% FBS, 0.1% sodium azide, and 0.2% saponin. Antibodies were then added, and cells were incubated for 20 min at 4 °C. For intracellular staining, cells were stimulated for 4 h with 50 ng/mL phorbol-12-myristate-13-acetate PMA (Sigma-Aldrich, San Luis, MO, USA), 500 ng/mL ionomycin (Sigma-Aldrich, San Luis, MO, USA), and Golgi Stop (BD Bioscience Franklin Lakes, Nova Jersey, EUA). The following antibodies were used: CD3ε (145-2C11), APC-Cy7; CD4 (RM4-5), PerCP; CD8α; FITC; IL-17 (TC11-18H10), Alexa Fluor 647 and PE; and IFN-γ (557998), Alexa Fluor 700. Cells were analyzed using a FACS Canto II flow cytometer, and the data were analyzed using the FlowJo (BD Bioscience Franklin Lakes, Nova Jersey, EUA) software.

### 2.4. Detection of Cytokine Levels in Pancreatic and Ileum Tissues

Pancreatic (tail portion) and ileum fragments were removed, weighed, and placed in a tube containing 700 µL of a complete protease inhibitor cocktail (Roche Diagnostics, Abbott Park, IL, USA). The pancreatic tissue fragments were removed at 0 and 15 days after the STZ injections. Ileum fragments were removed at 0, 7 and 15 days after the STZ injections. The tissues were homogenized using a Polytron homogenizer (Thermo Fisher Scientific, Waltham, MA, USA). The IL-1β, IL-12p70, IL-17, IFN-γ and IL-18 levels were detected by ELISA using colorimetric kits, according to the manufacturer’s instructions (R&D Systems). The results were expressed as the mean ± SEM, nanograms per gram of tissue (ng/g, pancreatic tissue) or picograms per milliliter (pg/mL), in the culture supernatant.

### 2.5. Quantification of Serum Insulin Levels

The serum samples were collected 15 days after MLD-STZ administration, and the insulin concentrations were determined using the Mouse Ultrasensitive Insulin ELISA kit (Alpco Diagnostics, 26-G Keewaydin Drive, Salem, NH 03079), according to the manufacturer’s instructions.

### 2.6. Histological and Immunohistochemistry Analysis

Pancreatic fragments (head portion) were removed, fixed in 10% buffered formalin, and embedded in paraffin. Then, 4–5 µm sections were stained with hematoxylin and eosin (Merck, Whitehouse Station, NJ, USA). Immunohistochemistry reactions were performed as previously described [31]. The degree of insulitis was evaluated using a semiquantitative scale: 0, intact islet; 1, peri-insulitis; 2, moderate insulitis (<50% of the islets infiltrated); and 3, severe insulitis (>50% of the islets infiltrated).

### 2.7. Western Blotting

Fifty micrograms of extracted proteins were directly loaded into a sodium dodecyl sulfate (SDS) sample buffer for 10% SDS-polyacrylamide gel electrophoresis. After transferring the samples onto a nitrocellulose membrane (Trans-Blot Transfer Medium; Bio-Rad, Hercules, CA, USA), the membranes were blocked with 5% milk in a Tris buffer solution containing 0.1% Tween 20 for 1 h and then incubated with antibodies against Capase-1 (Santa Cruz Biotechnology, Dallas, TX, USA, EUA) or AIM2 (Cell signaling, Cat. 12498S, Danvers, MA, USA) overnight at 4 °C. Next, the cells were incubated with an IgG horseradish peroxidase (HRP)-conjugated secondary Ab (Cell Signaling, Cat.7076S, Danvers, MA, USA) for 1 h at room temperature. After the membranes were rinsed, the immunocomplexes were developed using an enhanced peroxidase/luminol chemiluminescence reaction (ECL Western blotting detection reagents, Pierce Biotechnology, Waltham, MA, USA) and exposed to an X-ray film with autoradiography (Carestream Health, Rochester, NY, USA). The bands were quantified densitometrically using the ImageTool 2.0 software (University of Texas, Austin, TX, USA), and the results were expressed as arbitrary units.

### 2.8. Bone Marrow-Derived Macrophage (BMDM) Cultures and Stimulation

The BMDMs from the WT and AIM2^−/−^ mice were differentiated as previously described [32]. Briefly, the total bone marrow cells were cultured for 7 days in an RPMI 1640 medium (Sigma-Aldrich), supplemented with 10% fetal bovine serum (FBS) (Life Technologies, Molecular Probes, Carlsbad, CA, USA) and 30% L-929 cell-conditioned media at 37 °C and 5% CO_2_. Cells (0.5 × 10^6^/well) were pre-stimulated with lipopolysaccharide (LPS, 50 μg) for 4 h and stimulated for 6 h with Poly dA: dT (1.5 μg/mL) (AIM2 agonist), control fecal DNA (1.5 μg/mL) or fecal DNA from a diabetic animal (1.5 μg/mL). Subsequently, the IL-18 and IL-1β levels were determined in the supernatant of the cultured cells using the ELISA method.

### 2.9. RNA Extraction and Quantitative RT-PCR

The total RNA was extracted from the PLNs or pancreatic tissue using Trizol (Life Technologies, Molecular Probes, Carlsbad, CA, USA), following the manufacturer’s instructions. cDNA was obtained using a high-capacity reverse transcription kit (Applied Biosystems, Foster City, CA, USA). Quantitative mRNA analysis by RT PCR was performed using the SYBR Green fluorescence system (Applied Biosystems). The specific mRNA expression levels were normalized relative to the β2 microglobulin levels using the comparative 2^−ΔΔCt^ method.

### 2.10. DNA Extraction and 16S rRNA Gene PCR Analysis

DNA extraction from the pancreatic PLNs was performed using a DNeasy Blood and Tissue kit (QIAGEN, Germantown Road Germantown, MD, USA), following the manufacturer’s instructions. For the PCR analysis, 10 ng of DNA and 1 µM of the 16S rRNA primer (forward, 5′-AACAGGATTAGATACCCTGGTAG-3′; reverse, 5′-GGTTCTTCGCGTTGCATC-3′) were used.

### 2.11. Intestinal Permeability by FITC-Dextran

After 12 h of fasting, the mice received fluorescein isothiocyanate (FITC)-dextran by gavage (250 mg/kg) (Sigma-Aldrich, San Luis, MO, USA). After 4 h, 0.5 mL of the blood samples were collected from the orbital plexus. The blood was centrifuged at 4 °C at 1000× *g* for 3 min. The serum was diluted in the same volume of PBS (pH 7.4) to analyze the FITC-dextran concentrations at an excitation wavelength of 485 nm and emission wavelength of 535 nm.

### 2.12. Antibiotic Treatment

The mice were administered daily doses of 1.86 mg ampicillin (Sigma-Aldrich), 0.96 mg vancomycin (Sigma-Aldrich), 1.86 mg neomycin sulfate (Sigma-Aldrich), and 1.86 mg metronidazole (Sigma-Aldrich), diluted in 300 µL of drinking water, by gavage for 21 days, before the first administration of MLD-STZ.

### 2.13. Immunofluorescence

Frozen sections were incubated with rabbit monoclonal anti-ZO-1 (ABCAM), followed by incubation with anti-rabbit IgG conjugated to Alexa 594 (1:400), Alexa 488, and Alexa 647 (1:400) (Abcam, Cambridge, MA, USA). The sections were stained with 4′,6-diamidino-2-phenylindole (DAPI) (Life Technologies, Molecular Probes, Carlsbad, CA, USA). Fluorescent images were collected using confocal Leica SP5 microscopy.

### 2.14. Statistical Analysis

The data were expressed as the mean ± standard deviation (SD). The differences observed among the several experimental groups were analyzed by applying one-way ANOVA, followed by the parametric Tukey’s test to compare multiple groups or Student’s *t*-test to compare two groups. All analyses were performed using the Prism 5.0 software (GraphPad Software, San Diego, CA, USA). The statistical significance was set at *p* < 0.05 h.

## 3. Results

### 3.1. AIM2 Receptor Expression during the Course of T1D in the Murine Model and Humans

**Figure 1:**
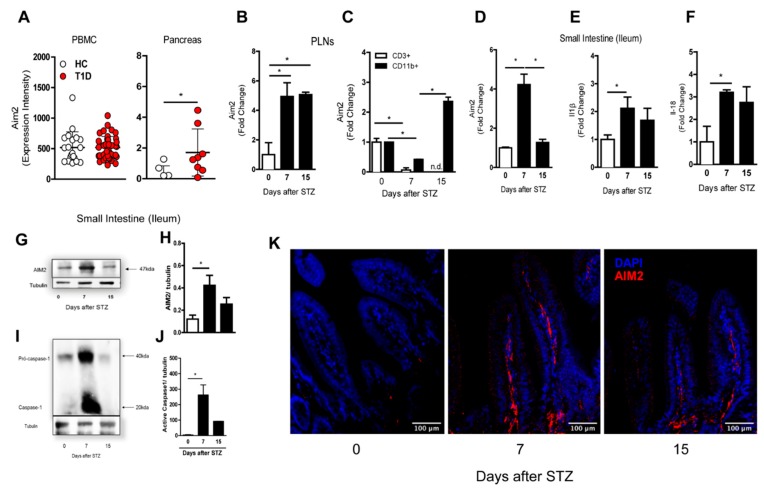
Expression of the AIM2 receptor in the pancreatic lymph nodes (PLNs) and small intestine in the streptozotocin STZ-induced type 1 diabetes (T1D) model. WT mice were injected with STZ (40 mg/kg) or a vehicle solution (VH, non-diabetic, 0 days after STZ) for five consecutive days. The PLNs and small intestine were recovered at 0, 7 and 15 days after the STZ injections. (**A**) The gene expression of Aim2 in the PBMC and pancreatic tissue of T1D patients, obtained from the Gene Expression Omnibus (GSE9006/GSE72492). The gene expression of Aim2 in (**B**) the PLNs by qPCR and (**C**) CD3+ lymphoid and CD11b+ myeloid cells, isolated by FACS from the PLNs from the WT diabetic or nondiabetic mice at 0, 7 and 15 days after the STZ injections. (**D**–**F**) The gene expression of Aim2, pro-il1β and pro-il18 in the small intestine of the WT mice at 0, 7 and 15 days after the STZ injections, respectively. (**G**–**J**) Western blot analysis of the AIM2 and active caspase-1 expression in the small intestine of the WT mice at 0, 7 and 15 days after the STZ injections. (**K**) Immunofluorescence microscopy of AIM2 in the small intestine of the WT mice at 0, 7 and 15 days after the STZ injections (AIM2 staining is represented in red, and DAPI staining is represented in blue). The values are expressed as the mean ± SD. * *p* < 0.05 was considered statistically significant, when compared with the WT mice at time 0 after the STZ injections. *N* = 3–6 animals per group. Significant differences between the groups were determined by one-way ANOVA, followed by Tukey’s multiple-comparison test.

First, we investigated alterations of the AIM2 expression in human subjects using the public gene-expression datasets available at the gene express omnibus (GSE9006/GSE72492) [33]. We found that AIM2 transcripts were increased in the pancreatic tissue of T1D patients, when compared to healthy controls (HC), but no significant alterations were observed in the peripheral blood mononuclear cell (PBMCs) of T1D patients, compared to HC (Figure 1A).

Accordingly, we found an increased gene expression of Aim2 in the PLNs of diabetic mice, 7 and 15 days after multiple low doses of streptozotocin (MLD-STZ), compared to non-diabetic mice (0 days) (Figure 1B). In addition, the AIM2 gene expression was upregulated only in myeloid, but not in lymphoid cells, 15 days after the STZ injections (Figure 1C). We also evaluated the gene and protein expression of Aim2, caspase-1, proIl1β and proIl18 in the small intestine (ileum) of STZ-injected mice. At day 7 after STZ induction, we found an increased Aim2 gene and protein expression. Increased active caspase-1 protein levels, as well as proIl1b and proIl18 mRNA levels, were also found (Figure 1D–J). Increased staining of the AIM2 protein, determined by immunofluorescence microscopy, was detected in the small intestine (ileum) of STZ-injected WT mice at day 7 after STZ induction (Figure 1K). The AIM2 staining in the small intestine at 15 days after the STZ injections was reduced, which is in agreement with the data described in Figure 1D,H. Overall, these results demonstrate that the expression and activation of the AIM2 receptor are increased in the PLNs and small intestine during an STZ-induced T1D course.

### 3.2. Deficiency of AIM2 Increases STZ-Induced T1D

**Figure 2:**
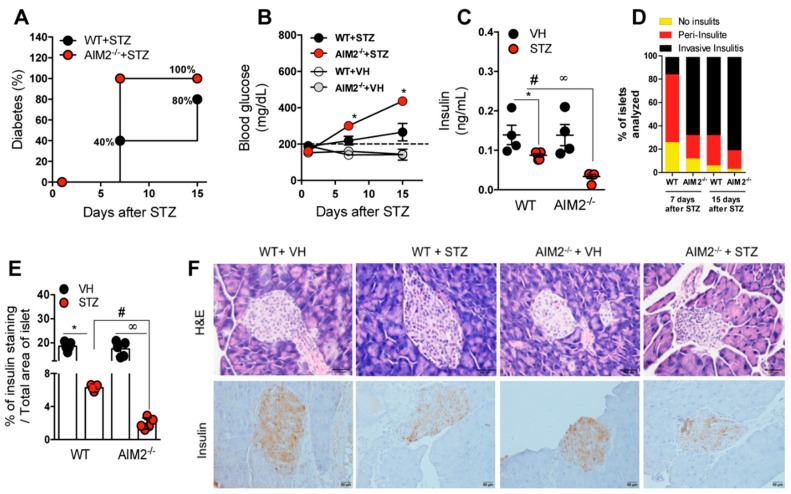
Deficiency of the AIM2 receptor accelerates Type 1 diabetes onset. The WT and AIM2^−/−^ mice were injected with STZ (40 mg/kg) or a vehicle solution (VH, non-diabetic, 0 days after STZ) for five consecutive days, and clinical parameters were evaluated. (**A**) Incidence of diabetes. (**B**) Blood glucose levels in the WT and AIM2^−/−^ mice at 0, 7 and 15 days after the STZ injections. (**C**) Insulin levels assessed by ELISA in the serum of the WT and AIM2^−/−^ mice at 15 days after the STZ injections. (**D**) Score of inflammatory infiltrates in the islets of the WT and AIM2^−/−^ mice at 7 and 15 days after the STZ injections. (**E**) Quantification of the insulin staining in the pancreatic tissue of the WT and AIM2^−/−^ mice at 15 days after the STZ injections. (**F**) Histopathology and insulin staining, assessed by immunohistochemistry in the pancreatic tissue of the WT and AIM2^−/−^ mice at 15 days after the STZ injections. The values are expressed as the mean ± SD. The results are considered statistically significant when *p* < 0.05 (*; #, ∞). *N* = 3–6 animals per group. Significant differences between the groups were determined by one-way ANOVA, followed by Tukey’s multiple-comparison test.

To further explore whether AIM2 has an effect in T1D development, we used the MLD-STZ model in the WT and AIM2^−/−^ mice, and the disease incidence and clinical parameters were monitored. Surprisingly, the AIM2^−/−^ mice were more susceptible to STZ-induced T1D, when compared to the WT mice, since 100% of the former developed the disease earlier, at day 7 after STZ induction, and, as expected, neither the WT nor the AIM2^−/−^ mice became diabetic after the vehicle injection (Figure 2A). Notably, the AIM2^−/−^ mice displayed a significant increase in glucose levels from day 7 to day 15, compared with levels in the WT mice after the STZ injection (Figure 2B). Additionally, the AIM2^−/−^ mice presented significant lower serum levels of insulin, compared to the WT mice injected with STZ (Figure 2C). Then, we verified whether the susceptibility of the AIM2^−/−^ mice could be attributed to the increased severity of the proinflammatory response in pancreatic islets. In fact, histological analysis showed that the STZ-injected AIM2^−/−^ mice exhibited an increased inflammatory infiltration (invasive insulitis) into the pancreatic islets at 7 and 15 days after STZ induction (Figure 2D,F top panels). Consistent with these results, the STZ-injected AIM2^−/−^ mice showed decreased insulin staining in pancreatic islets, compared to the WT mice injected with STZ (Figure 2E,F bottom panels).

These findings suggest that the AIM2 receptor attenuates the inflammatory response and limits the damage of the insulin-producing β cells of pancreatic islets, resulting in resistance to STZ-induced T1D.

### 3.3. AIM2 Receptor Activation Dampens the Pathogenic Th1/Tc1 Cell Response in STZ-Induced T1D

**Figure 3:**
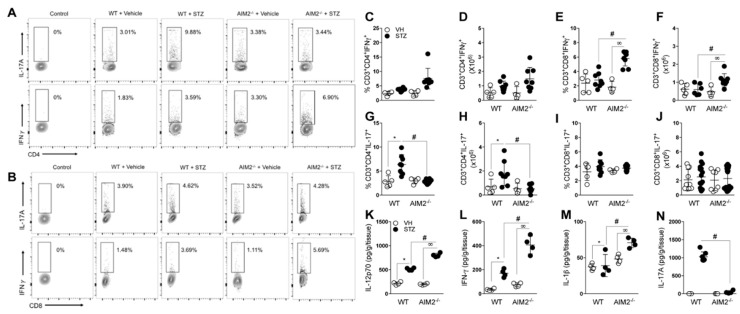
Deficiency of AIM2 increases Th1 and Tc1 populations in PLNs during T1D. Cells from the PLNs of the WT and AIM2^−/−^ mice were harvested at 15 days after the vehicle (VH) or STZ administration and assessed for extracellular (CD3, CD4 and CD8) and intracellular (IL-17 and IFN-γ) molecules, with a minimum of 2 × 10^6^ cells per sample. (**A**,**B**) Representative dot plot of CD4^+^IL-17^+^ (Th17), CD8^+^IL-17^+^ (Tc17), CD4^+^ IFN-γ^+^ (Th1), and CD8^+^ IFN-γ^+^ (Tc1) cells in the PLNs of the WT and AIM2^−/−^ mice, after the STZ injections. (**C**–**J**) Percentage and absolute numbers of CD4^+^ IFN-γ^+^ (Th1), CD4^+^IL-17^+^ (Th17), CD8^+^IL-17^+^ (Tc17), and CD8^+^ IFN-γ^+^ (Tc1) cells in the PLNs of the WT and AIM2^−/−^ mice, after 0, 7 and 15 days of the STZ injections. (**K**–**N**) Protein levels of IL-12p70, IFN-γ, IL-1beta and IL-17 cytokines, assessed by ELISA in the pancreatic tissue of the WT and AIM2^−/−^ mice, after 15 days of STZ or vehicle injections. The values are expressed as the mean ± SD. The results were considered statistically significant when *p* < 0.05 (*; #, ∞). *N* = 3–6 animals per group. Significant differences between the groups were determined by one-way ANOVA, followed by Tukey’s multiple-comparison test.

Considering that the activation of the AIM2 receptor may have a protective role in the STZ-induced T1D model, we next investigated whether AIM2 deficiency interferes with the pathogenic T cells population in vivo. Our results show that the AIM2^−/−^ mice exhibited a higher frequency of Th1 and Tc1 cells (CD4^+^ and CD8^+^ producing IFN-γ T cells) and increased the absolute numbers of Tc1 cells in the PLNs (Figure 3A–F). In parallel, we detected increased levels of IL-12p70, IL-1β and IFN-γ cytokines in the pancreatic tissue of the AIM2^−/−^ mice, compared to the diabetic WT, at 15 days after the STZ injections (Figure 3K,L). Nevertheless, the deficiency of AIM2 significantly decreased the frequency and absolute numbers of Th17 (CD4^+^ producing IL-17 T cells), but not those of Tc17 (CD8^+^ producing IL-17 T cells) cells, in the PLNs at the same time (Figure 3A,B, and G–J). Accordingly, we found a significant reduction in IL-17 levels in the pancreatic tissue (Figure 3M,N), suggesting an involvement of AIM2 in the generation of Th17 cells during STZ-induced T1D.

Collectively, these results suggest that the AIM2 receptor negatively regulates the generation of pathogenic Th1 and Tc1 cell response in PLNs during STZ-induced T1D development.

### 3.4. AIM2 Receptor Controls Gut Microbiota Translocation to PLNs in STZ-Induced T1D

**Figure 4:**
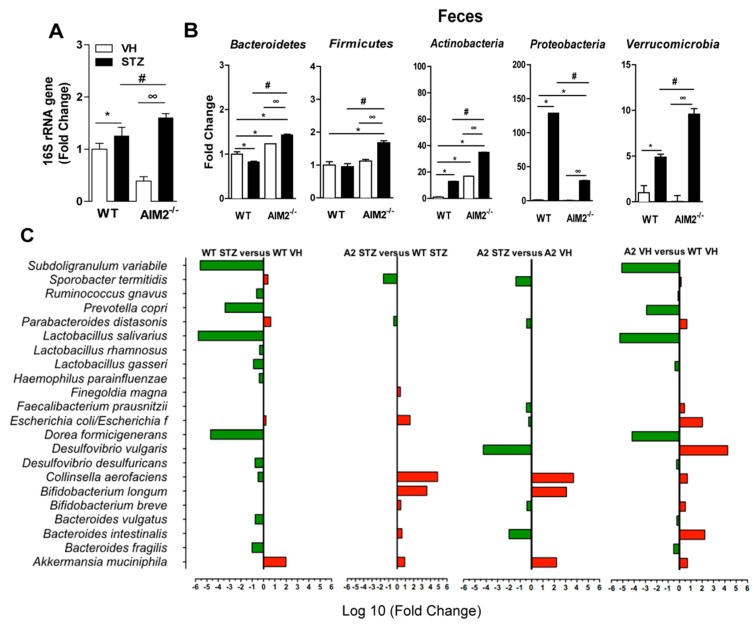
AIM2 receptor controls gut microbiota translocation to PLNs during T1D. The vehicle (VH)- or STZ-injected WT and AIM2^−/−^ mice were killed, and the PLNs were collected for the analysis of bacterial translocation by the 16S rRNA gene expression using qPCR. (**A**) The 16S rRNA gene expression in the PLNs of the WT and AIM2^−/−^ mice at 0 and 15 days after the STZ injections. (**B**) The gene expression of the bacteria from Bacteroidetes, Actinobacteria, Firmicutes, Proteobacteria and Verrucomicrobia phyla in the feces of the WT and AIM2^−/−^ mice at 15 days after the injection of the vehicle or STZ. (**C**) PCR array representing the gene expression of the bacterial species in the feces of the WT and AIM2^−/−^ mice at 15 days after the injection of vehicle or STZ. The values are expressed as the mean ± SD or log10 (fold). The results are considered statistically significant when *p* < 0.05 (*; #, ∞). *N* = 3–6 animals per group. Significant differences between the groups were determined by one-way ANOVA, followed by Tukey’s multiple-comparison test.

Our group recently demonstrated that gut microbiota translocation to PLNs triggers a proinflammatory response and contributes to damage of insulin-producing β cells and T1D onset [21]. Moreover, previous reports have shown that the AIM2 receptor plays an important role in intestinal homeostasis and controls gut microbiota translocation [29,30]. Considering the increased AIM2 expression in the small intestine at 7 days after the STZ injection and the increased proinflammatory response in the PLNs of the AIM2^−/−^ diabetic mice, we hypothesized that AIM2 controls gut microbiota translocation to PLNs during T1D and prevents the development of a proinflammatory response against insulin-producing β cells. Interestingly, we detected an increased expression of the bacterial 16S rRNA gene in the PLNs of the AIM2^−/−^ mice at 15 days after the STZ injections (Figure 4A), which suggests that the lack of AIM2 increases gut microbiota translocation to PLNs during T1D. Given the fact that gut microbiota dysbiosis is associated with bacterial translocation in T1D development, both in humans and mice [9,11,14,21,34,35], we next assessed whether diabetic AIM2^−/−^ mice have gut microbiota with an altered composition. First, we observed a significant increase in the abundance of Bacteroidetes, Firmicutes, Actinobacteria, and Verrucomicrobia bacterial phyla, but a significant decrease in Proteobacteria, in the feces of the AIM2^−/−^ mice, compared to the WT mice (Figure 4B). In agreement, the abundance of Bacteroidetes phylum was significantly increased in the PLNs of the AIM2^−/−^ diabetic mice (data not shown).

Next, we performed a PCR array to determine the abundance of the bacterial species that were altered in the feces of the WT and AIM2^−/−^ mice, both in healthy and diabetic conditions. The analysis of the abundance of bacterial species in the feces of STZ-injected WT mice, compared to the Vehicle-injected WT mice, revealed a significant decrease in the abundance of *Lactobacillus salivarius, Subdoligranulum variabile*, *Prevotella copri, Dorea formicigerans, Bacteroidetes fragilis and Bacteroidetes vulgatus*, but also an increased abundance of *Akkermansia muciniphila* and *Parabacteroides distasonis*. Interestingly, the STZ-injected AIM2^−/−^ mice, compared to the STZ-injected WT mice, showed an increased abundance (log 10-fold) of various bacterial species, especially *Collinsella aerofaciens.* In a lower proportion, we also detected an increase in the abundance of *Bifidobacterium longum, Escherichia coli, Akkermansia muciniphila* and *Bacteroides intestinali* in the feces of these mice. In parallel, we also observed a decreased expression of *Sporobacter termiditis* and *Parabacteroides distasonis* in the feces of the AIM2^−/−^ mice (Figure 4C).

The analysis of the abundance of bacterial species in the feces of the Vehicle-injected AIM2^−/−^ mice, compared to the Vehicle-injected WT mice, revealed a decreased expression of some bacterial strains, such as *Subdoligranulum variable, Prevotela copri, Lactobacilus salivarus, Haemophilus parainfluenza, Dorea formicigenerans and Bacteroides intestinalis.* However, the Vehicle-injected AIM2^−/−^ mice showed an increased expression of *Bacteroidetes intestinalis*, *Desulfovibrio vulgaris*, *Escherichia coli* and *Parabacteroides distasonis*. Finally, we noted that the AIM2^−/−^ mice, after STZ administration, compared to the Vehicle-injected AIM2^−/−^ mice, also exhibited an increased abundance of *Collinsella aerofaciens, Bifidobacterium longum,* and *Akkermansia muciniphila* and a decreased expression of *Bacteroides intestinalis, Desulfovibrio vulgaris, Bifidobacterium breve* and *Sporobacter termiditis* (Figure 4C).

Overall, our results show that: (i) STZ increases bacterial translocation to the PLNs in both WT and AIM2^−/−^ mice; and (ii) a deficiency of AIM2 is associated with changes in the gut microbiota, which, in turn, may be linked to an increased T1D severity.

### 3.5. Antibiotic Treatment Abrogates Bacterial Translocation and the Generation of a Proinflammatory Response in STZ-Induced T1D

**Figure 5:**
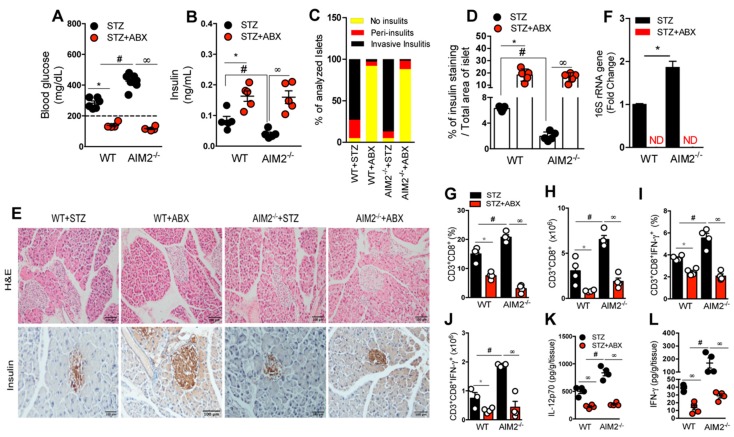
AIM2 receptor restrains gut microbiota translocation and controls the generation of a proinflammatory response in PLNs during T1D. The WT and AIM2^−/−^ mice were treated with an antibiotic cocktail (ABX) for 21 days before the STZ injections, and the clinical and immunological parameters were determined. (**A**) Blood glucose and (**B**) insulin levels in the serum of the WT and AIM2^−/−^ mice at 15 days after the vehicle and STZ injections. (**C**) Score of the inflammatory infiltrates in the islets of the WT and AIM2^−/−^ mice at 15 days after the vehicle and STZ injections or after the ABX and STZ injections. (**D**) Quantification of insulin in the pancreatic tissue of the WT and AIM2^−/−^ mice at 15 days after the STZ injections. (**E**) Histopathology and insulin staining, assessed by immunohistochemistry, in the pancreatic tissue of the WT and AIM2^−/−^ mice at 15 days after the vehicle and STZ injections or after the ABX and STZ injections. (**F**) The 16S rRNA gene expression in the PLNs of the WT and AIM2^−/−^ mice at 15 days after the STZ injections. (**G**–**J**) The percentage and absolute numbers of CD3^+^CD8^+^ and CD3^+^CD8^+^IFN-γ^+^ cells in the PLNs of the WT and AIM2^−/−^ mice at 15 days after the STZ injections. (**K**,**L**) The protein levels of IL-12 p70 and IFN-γ cytokines, assessed by ELISA, in the pancreatic tissue of the WT and AIM2^−/−^ mice at 15 days after the STZ injections. The values are expressed as the mean ± SD. The results are considered statistically significant when *p* < 0.05 (*; #, ∞). *N* = 3–6 animals per group. Significant differences between the groups were determined by one-way ANOVA, followed by Tukey’s multiple-comparison test.

To assess whether gut microbiota translocation is related to increased inflammation in the absence of AIM2, resulting in a higher risk of T1D development after the STZ injections, the WT and AIM2^−/−^ mice were subjected to a daily antibiotic cocktail pretreatment (ABX: metronidazole, vancomycin, ampicillin, and neomycin) for 21 days, before the STZ injections, in order to deplete the gut microbiota. Then, T1D was induced, and the clinical parameters were determined. Interestingly, both the WT and AIM2^−/−^ mice that received the ABX presented lower blood glucose levels and were protected from T1D development, when compared with the mice that did not receive ABX but only the STZ injections (Figure 5A).

The ABX administration also increased serum insulin levels and the insulin expression in the pancreatic islets of both the STZ-injected WT and AIM2^−/−^ mice (Figure 5B,D,E, bottom panels). In addition, the ABX administration reduced the pancreatic islet inflammation in the STZ-treated mice, relative to the non-treated mice (Figure 5C,E, top panels). Interestingly, we did not detect the 16S rRNA bacterial gene expression in the PLNs of the AIM2^−/−^ mice, after the ABX and STZ injections (Figure 5F). Additionally, the ABX treatment reduced the frequency and absolute numbers of IFN-γ-producing CD8^+^ T cells (Tc1) in PLNs, as well as significantly downregulated the levels of IL-12p70 and IFN-γ in the pancreatic tissue of the AIM2^−/−^ mice, after the STZ injection, compared to the non-treated mice (Figure 5G–L).

Taken together, our results suggest that the increased gut microbiota translocation in the STZ-injected AIM2^−/−^ mice may be responsible for the increased susceptibility of these mice to T1D.

### 3.6. AIM2 Receptor Controls Intestinal Permeability through the Regulation of Tight-Juntion Proteins in STZ-Induced T1D

**Figure 6:**
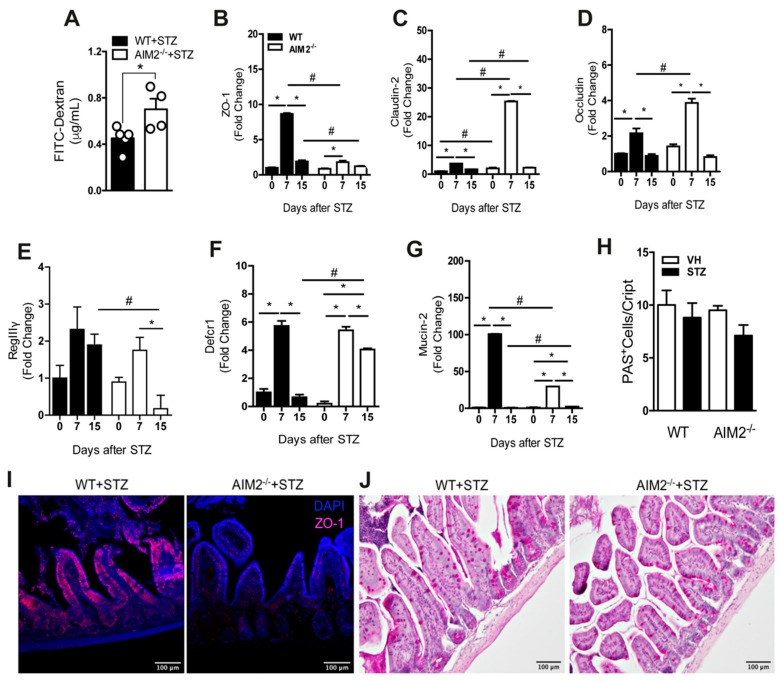
AIM2 receptor regulates the intestinal barrier function to control gut permeability during T1D. (**A**) FITC-dextran levels in the serum of the WT and AIM2^−/−^ mice at 15 days after the STZ injections. (**B**–**G**) The gene expression of ZO-1, claudin-2, occludin, RegIIIγ, Defcr1, and mucin-2, respectively, in the small intestine of the WT and AIM2^−/−^ mice at 0, 7 and 15 days after the STZ or vehicle (VH) injections. (**I**) Immunofluorescence microscopy representing the ZO-1 (purple), WT and AIM2^−/−^ mice at 7 days after the STZ injections. (**H**,**J**) Periodic acid–Schiff (PAS) staining in the small intestine of the WT and AIM2^−/−^ mice at 15 days after the STZ injections. The values are expressed as the mean ± SD. The results are considered statistically significant when *p* < 0.05 (*; #, ∞). *N* = 3–6 animals per group. Significant differences between the groups were determined by one-way ANOVA, followed by Tukey’s multiple-comparison test.

Alterations in gut permeability are associated with gut microbiota translocation in several diseases, including T1D [15,36,37,38,39]. Since the AIM2^−/−^ mice displayed an increased gut microbiota translocation to PLNs after the STZ injections, we next assessed whether the AIM2 receptor regulates intestinal permeability and, consequently, controls the translocation to PLNs. First, we performed an FITC-dextran assay to determine the intestinal permeability in the WT and AIM2^−/−^ mice, after the STZ injections. We detected significantly increased levels of FITC-dextran in the serum of the AIM2^−/−^ mice at 15 days after the STZ injections, when compared to the WT mice (Figure 6A), suggesting that the intestinal barrier of the AIM2^−/−^ mice is disrupted during T1D.

Considering that tight-junction proteins are involved in the regulation of intestinal permeability [40,41,42,43], we next evaluated whether AIM2 deficiency alters the gene expression of tight-junction proteins, such as zonnulin-1 (ZO-1), claudin-2, and occludin, after the STZ injections. Interestingly, the gene expression of ZO-1, claudin-2 and occludin was significantly increased at 7 days after the STZ injections in the ileum of the WT mice. On the other hand, the ZO-1 gene expression was reduced, whereas the claudin-2 and occludin expression was significantly increased in the ileum of the AIM2^−/−^ mice at 7 days after the STZ injections (Figure 6B–D). We also observed a lower staining of the ZO-1 protein in the ileum of the AIM2^−/−^ mice at 7 days after the STZ injections (Figure 6I). Moreover, the mucin-2 and RegIIIγ expression was significantly decreased in the ileum of these mice at 7 and 15 days after the STZ injections, respectively (Figure 6E,G). On the other hand, the AIM2^−/−^ mice exhibited a significant increase in the Defcr1 expression in the ileum at day 15, compared to the WT mice, after the STZ injections (Figure 6F). Despite a trend toward decreased mucus staining in the ileum of the AIM2^−/−^ mice at 15 days after the STZ injections, compared to the WT mice, no significant differences were observed (Figure 6H,J).

Taken together, our results suggest that the AIM2 receptor is required for the maintenance of the intestinal barrier through the regulation of tight-junction proteins and the RegIIIγ expression by intestinal epithelium during T1D.

### 3.7. Deficiency of AIM2 Impairs IL-18 and IL-1 β Production in the Gut Mucosa during STZ-Induced T1D

**Figure 7:**
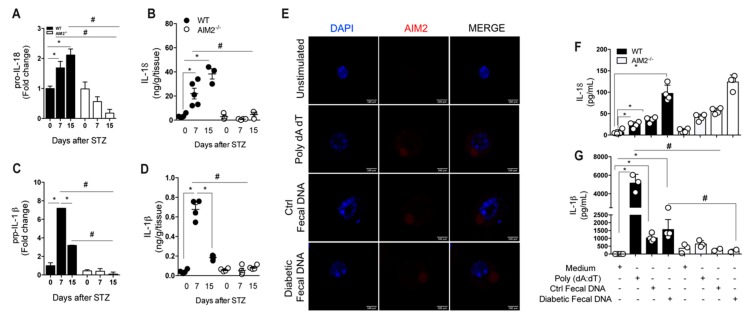
Deficiency of AIM2 impairs IL-18/IL-1β production in the gut mucosa during STZ-induced T1D. (**A**,**B**) The gene and protein expression of IL-18 in the ileum of the WT and AIM2^−/−^ mice at 0, 7 and 15 days after the STZ induction. (**C**,**D**) The gene and protein expression of IL-1β in the ileum of the WT and AIM2^−/−^ mice at 0, 7 and 15 days after the STZ induction. (**E**) Immunofluorescence of AIM2 in WT bone marrow-derived macrophages (BMDM), after 18 h of stimulation with the medium (unstimulated), Poly dA dT (AIM2 agonist), control fecal DNA, and diabetic fecal DNA. (**F**,**G**) The protein expression, assessed by ELISA, of the IL-18 and IL-1ß of the WT and AIM2^−/−^ BMDM, after 18 h of stimulation with the medium (unstimulated), Poly dA dT (AIM2 agonist), control fecal DNA, and diabetic fecal DNA. The values are expressed as the mean ± SD. The results are considered statistically significant when *p* < 0.05 (*; #, ∞). *N* = 3–6 animals per group. Significant differences between the groups were determined by one-way ANOVA, followed by Tukey’s multiple-comparison test.

Previous studies have shown that AIM2 activation leads to the release of IL-18 and IL-1β and contributes to the maintenance of intestinal homeostasis [29,30]. In line with these studies, we found a decreased mRNA and protein expression of proIL-18 and proIL-1β in the ileum of the AIM2^−/−^ mice at 7 and 15 days after the STZ injections, compared with the WT diabetic mice (Figure 7A–D).

These results demonstrate that a deficiency of AIM2 impairs IL-18 and IL-1β production, suggesting that AIM2 activates the inflammasome platform. Ratsimandresy et al. (2017) demonstrated that AIM2 activation by gut microbiota DNA induces both IL-18 and IL-1β production by BMDM. Next, we evaluated whether gut microbiota DNA from diabetic and healthy mice activates AIM2 inflammasome in macrophages in vitro. First, we used AIM2 citrin macrophages, which constitutively express AIM2 (AIM2 citrine), and WT and AIM2^−/−^ BMDMs. The macrophages were stimulated with either the AIM2 agonist, Poly dAdT, gut microbiota DNA isolated from a healthy mouse (Ctrl fecal DNA), or gut microbiota DNA isolated from diabetic mice (Diabetic fecal DNA). Afterwards, we evaluated the expression and co-localization of AIM2 upon exposure to fecal DNA (Ctrl and diabetic). Interestingly, we observed that AIM2 co-localizes and forms specks with Poly dA dT, control fecal DNA and diabetic fecal DNA in AIM2 citrine macrophages, suggesting that both DNA are recognized by AIM2 (Figure 7E). Additionally, the levels of IL-18 in the supernatant of WT BMDMs were increased after stimulation with Poly dA dT and control fecal DNA, but more markedly after stimulation with diabetic fecal DNA, compared to BMDMs kept in the medium only (Figure 7F). On the other hand, WT BMDMs produced high levels of IL-1β, after stimulation with Poly dA dT, but the increased levels of IL-1β were less pronounced, after the stimulation of WT BMDMs with diabetic fecal DNA or ctrl fecal DNA (Figure 7G). Surprisingly, the BMDMs from AIM2-deficient mice exhibited a significant reduction in IL-1β levels but similar IL-18 levels, after stimulation with Poly dA dT or diabetic fecal DNA, compared to the BMDMs from the WT mice (Figure 7F,G). Overall, our data suggest that AIM2 activates the inflammasome in vivo and leads to IL-18 and IL-1β release in gut mucosa during T1D development. Gut microbiota DNA from diabetic mice remarkably induces IL-18 release in vitro, although our data do not show that IL-18 production depends on AIM2 receptor activation.

### 3.8. IL-18 Is Required for the Maintenance of Intestinal Homeostasis during T1D

**Figure 8:**
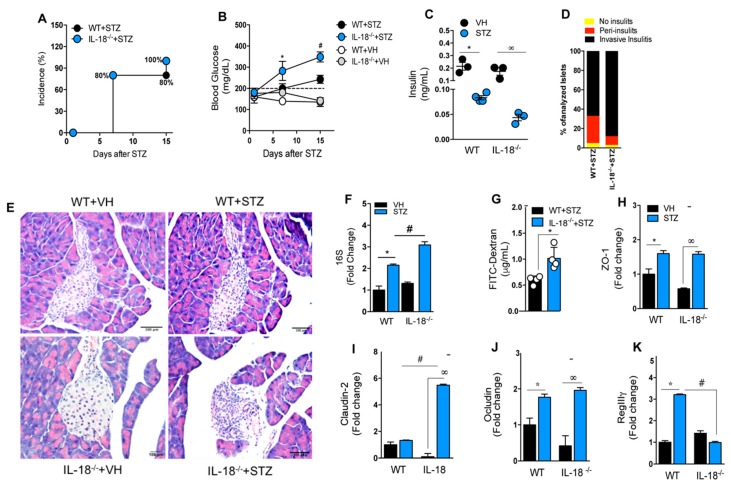
Deficiency of IL-18 increases intestinal permeability and accelerates Type 1 diabetes onset. The WT and IL-18^−/−^ mice were injected with STZ (40 mg/kg) or a vehicle solution (VH, non-diabetic, 0 days after STZ) for five consecutive days, and the clinical parameters were evaluated. (**A**) The incidence of diabetes. (**B**) The blood glucose levels in the WT and IL-18^−/−^ mice at 0, 7 and 15 days after the vehicle or STZ injections. (**C**) The insulin levels, assessed by ELISA, in the serum of the WT and IL-18^−/−^ mice at 15 days after the vehicle or STZ injections. (**D**) The score of inflammatory infiltrates in the islets of the WT and IL-18^−/−^ mice at 15 days after the vehicle or STZ injections. (**E**) Histopathology of the pancreatic tissue of the WT and IL-18^−/−^ mice at 15 days after the STZ injections. (**F**) The 16S rRNA gene expression in the PLNs of the WT and IL-18^−/−^ mice at 15 days after the STZ injection. (**G**) The FITC-Dextran levels in the serum of the WT and the IL-18^−/−^ mice at 15 days after the STZ or vehicle injections. (**H**–**L**) The gene expression of ZO-1, claudin-2, Occludin and RegIIIy, respectively, in the ileum of the WT and IL-18^−/−^ mice at 15 days after the STZ induction. The values are expressed as the mean ± SD. The results were considered statistically significant when *p* < 0.05 (*; #, ∞). *N* = 3–6 animals per group. Significant differences between the groups were determined by one-way ANOVA, followed by Tukey’s multiple-comparison test.

The results suggest that AIM2 protects against T1D by controlling intestinal permeability, which prevents gut microbiota translocation to PLNs and the generation of a pro inflammatory response. This phenotype is also associated with a reduced production of IL-18 and IL-1β in the ileum of the STZ-injected AIM2^−/−^ mice. Previous studies have shown that the production of IL-18 in the intestinal mucosa mediated by AIM2 activation contributes to the regulation of intestinal homeostasis [30]. Thus, we next hypothesized that IL-18 production by AIM2 inflammasome activation in the early stages of T1D contributes to intestinal homeostasis, preventing gut microbiota translocation and T1D susceptibility. Interestingly, we observed that IL-18^−/−^ mice, as well as AIM2^−/−^ mice, are more susceptible to T1D development, after STZ injections, compared to WT mice, showing a higher incidence of the disease, as well as elevated blood glucose levels and decreased insulin serum levels (Figure 8A–C). Similar to AIM2-deficient mice, IL-18^−/−^ mice displayed an increased inflammatory pancreatic infiltration (invasive insulitis) at 15 days after the STZ induction (Figure 8D). In addition, the bacterial 16S rRNA gene in the PLNs and intestinal permeability, assessed by FITC-dextran, were increased in the IL-18^−/−^ mice at 15 days after the STZ injections (Figure 8F,G). We also found that the IL-18^−/−^ mice had an increased expression of claudin-2 and decreased expression of REGIIIγ in the ileum at 15 days after the STZ injections, compared to the WT mice (Figure 8I,K). However, no significant differences in the expression of ZO-1 and occludin in the ileum of the WT and STZ-injected IL-18^−/−^ mice were found (Figure 8H,K).

Overall, our results suggest that IL-18 production regulates claudin-2 and REGIIIγ expression and controls intestinal permeability, possibly by AIM2 inflammasome activation in the small intestine, in order to limit gut microbiota translocation to PLNs, which protect against STZ-induced T1D development.

## 4. Discussion

In this study, we demonstrate the importance of the DNA sensor, AIM2, in the control of T1D development. AIM2 regulates intestinal homeostasis and limits gut microbiota translocation to PLNs, therefore controlling the development of a proinflammatory response against insulin-producing β cells. Notably, mice lacking the AIM2 receptor develop faster and more severe T1D, characterized by a higher blood glucose and decreased insulin serum levels as well as increased invasive insulitis during T1D. Furthermore, AIM2^−/−^ diabetic mice have increased proinflammatory response in PLNs and pancreatic tissue, which are associated with increased Th1 and Tc1 cell populations in PLNs and increased levels of IL-12p70, IFN-γ cytokines in the pancreas. Accordingly, AIM2^−/−^ diabetic mice have increased gut microbiota translocation to PLNs. Interestingly, when AIM2^−/−^ mice are treated with a broad-spectrum antibiotic cocktail that depletes gut microbiota, bacterial translocation is abrogated, and these mice become resistant to T1D, as observed in the WT mice, events associated with decreased inflammation in PLNs and pancreatic tissue. Investigating why AIM2^−/−^ diabetic mice exhibit greater bacterial translocation that leads to a proinflammatory response and T1D progression, we found that mice lacking AIM2 undergo changes in the intestinal barrier that are associated with a greater intestinal permeability and altered expression of tight-junction proteins.

The intestinal barrier disruption in the AIM2^−/−^ diabetic mice is associated with an increased expression of *Collinsella aerofaciens* bacteria in the feces and an impaired IL-18 production, which has been identified as a regulator of intestinal permeability [29,30]. Similar to the AIM2-deficient mice, the STZ-injected IL-18^−/−^ mice have higher blood glucose levels, decreased serum insulin, and increased invasive insulitis. Hyperglycemia and increased inflammation in STZ-injected IL-18^−/−^ mice are linked to an increased bacterial translocation to PLNs and increased intestinal permeability, suggesting that IL-18 production in the gut mucosa is important for maintaining the intestinal integrity in STZ-induced T1D, preventing gut microbiota translocation to PLNs and, consequently, inflammation. The susceptibility of AIM2^−/−^ mice to autoimmune/inflammatory disease was previously reported in a DSS-induced colitis model. As for T1D, AIM2^−/−^ mice are highly susceptible to DSS-induced colitis, displaying an increased loss of body weight, increased diarrhea score, and more severe colon inflammation [29,30]. These studies reported AIM2 as a crucial regulator of gut microbiota and intestinal homeostasis.

A recent finding of our group shows that commensal microbiota translocate from the gut to PLNs and activate a NOD2-mediated proinflammatory response, which contributes to T1D onset [21]. Additionally, gut microbiota translocation is also observed in NOD mice, before the onset of T1D [44], and is involved in the development of other autoimmune diseases, such as SLE [45] and cholangitis [46]. We found an increased bacterial translocation in the PLNs of the STZ-injected AIM2^−/−^ mice at 7 and 15 days after STZ induction, when compared to the WT diabetic mice. In addition, the AIM2^−/−^ diabetic mice displayed a significant increase in bacteria from the Bacteroidetes phylum in the PLNs at 15 days after STZ induction (data not shown). These results corroborate data from type 1 diabetes patients, with the *Bacteroidetes* phylum being more commonly detected in autoantibody-positive children than in autoantibody-negative peers [11]. We also found that STZ-injected AIM2^−/−^ mice displayed an increased fecal expression of *C. aerofaciens*, *Bifidobacterium longum*, *Escherichia coli* and *Bacteroides intestinalis*, when compared to STZ-injected WT mice. A recent study showed that the enrichment of the feces with *C. aerofaciens* and *B. longum* in melanoma patients, who received anti-PD1 treatment, is associated with an increased frequency of CD8^+^ T cells and reduced Foxp3^+^CD4^+^ Tregs in the tumor microenvironment [47]. The AIM2^−/−^ mice with T1D exhibited increased *C. aerofaciens* in their feces, which was associated with increased diabetogenic CD8^+^ T cells. Therefore, it is possible that the increased abundance of these bacteria in our murine T1D model is associated with an induction of diabetogenic CD8^+^ T cells in STZ-injected AIM2^−/−^ mice. Additionally, the relative abundance of *Collinsella* genus in the feces of Rheumatoid arthritis (RA) patients was correlated with higher levels of alpha-aminoadipic acid and asparagine. In accordance, the administration of *C. aerofaciens* in a humanized mouse model of RA increased the disease severity, which was associated with a decreased expression of the tight-junction proteins, ZO-1 and Occludin, as well as an increased gut permeability [48]. Moreover, the STZ-injected AIM2^−/−^ mice exhibited an increased fecal *E. coli* expression, which corroborates the data from the DSS-induced colitis model, with mice lacking AIM2 exhibiting more *E. coli* translocation to the colon and an increased susceptibility to colitis development [29].

We also tested whether the gut microbiota accounts for the T1D severity in STZ-injected AIM2^−/−^ mice by treating them with a broad-spectrum antibiotic cocktail (ABX) before the STZ injections. We found that both the WT and AIM2^−/−^ mice were completely protected from STZ-induced T1D development, with no bacterial translocation, reduced blood glucose levels, reestablishment of serum insulin levels, or decreased inflammatory response in the PLNs and pancreatic tissue. Similar results were found in the DSS-induced colitis model. Mice lacking AIM2, which were treated with ABX before being treated with DSS, exhibited less body weight loss, a decreased diarrhea score, less colon inflammation and, consequently, did not develop colitis. In addition, the production of the proinflammatory cytokines IL-6, KC, MIP2 and CCL2 was reduced to the baseline in antibiotic-treated WT and AIM2^−/−^ mice [29,30]. The depletion of gut microbiota by ABX treatment was recently reported in a SLE model, in which the treatment with vancomycin or ampicillin-suppressed *E. gallinarum* translocation to liver and the development of SLE [45]. Similar findings were also reported in a cholangitis model. The administration of antibiotics to mice with a primary biliary cholangitis-manifesting altered composition of gut microbiota significantly alleviated T-cell-mediated infiltration and bile duct damage [46]. Our findings suggest that the AIM2 receptor limits gut microbiota translocation to PLNs, controlling the generation of a proinflammatory response against insulin-producing β cells and STZ-induced T1D development.

Dysbiosis is related to an increased intestinal permeability, and these alterations are associated with changes in the expression of tight-junction proteins that modulate bacterial translocation from the lumen to the lamina propria (LP) [49,50]. In an SLE model, the translocation of *E. gallinarum* to the liver was associated with changes in the expression of the tight-junction (TJ) proteins, zonnulin-1, claudin-3, claudin-5, occludin and JAM-A [45]. Our study shows that STZ-injected AIM2^−/−^ mice have an increased intestinal permeability, determined by a FITC-dextran assay, as well as changes in the expression of the TJ proteins, ZO-1, claudin-2 and occludin in the small intestine. STZ-injected AIM2^−/−^ mice also display a reduced gene expression of the antimicrobial peptide, REGIIIγ, after 15 days of STZ. These results recapitulate what has been found in type 1 diabetes patients, who display gut microbiota dysbiosis, after the ingestion of sugars, and an increased intestinal permeability, indicating damage to the intestinal barrier [51]. Accordingly, we also verified a reduced ZO-1 expression in the small intestine of AIM2^−/−^ diabetic mice. Other studies demonstrated an increased zonnulin expression, as a marker of intestinal permeability, in type 1 diabetes patients [15,52]. Moreover, in the DSS-induced colitis model, mice lacking AIM2 also show a reduced antimicrobial peptide expression, associated with gut microbiota dysbiosis, bacterial translocation and increased colitis susceptibility.

Finally, we found that during T1D, STZ-injected AIM2^−/−^ mice have an impaired IL-18 and IL-1β production in the gut mucosa. Previous studies have shown that the production of IL-18 in the intestinal mucosa mediated by AIM2 activation contributes to the regulation of intestinal homeostasis [30]. Indeed, a deficiency of IL-18 replicates results found in AIM2^−/−^ mice during T1D, in which IL-18^−/−^ mice were shown to be more susceptible to STZ-induced T1D, displaying higher blood glucose levels and lower serum insulin levels. Additionally, as observed in AIM2^−/−^ mice, STZ-injected IL-18^−/−^ mice display alterations in intestinal permeability, which are associated with a higher expression of claudin-2 and decreased expression of RegIIIγ. These results corroborate findings in the colitis model, in which AIM2 inflammasome was shown to be activated in the colon, leading to IL-18 release [29] and the regulation of intestinal homeostasis through the induction of antimicrobial peptides and mucus production [30]. In addition, we observed that BMDMs stimulated in vitro with fecal DNA from diabetic mice markedly produces IL-18 and IL-1β, compared to unstimulated cells (BMDMs kept in medium). Despite the absence of statistical significance, diabetic fecal DNA also induced more IL-18 and IL-1β production in WT BMDMs, when compared to control fecal DNA. These were surprising results, given the fact that AIM2 recognizes dsDNA in a sequence-independent manner. Analysis of the frequency of CpG motifs in the genomes of commensal bacteria showed that species from Proteobacteria, Bacteroidetes and Actinobacteria phyla carry high counts of GTCGTT, which is an optimal motif to stimulate TLR9 receptor [53]. TLR9 activation by CpG induces the production of Type I interferons [54], and these cytokines promote AIM2 expression [55]. Based on these results, we hypothesize that diabetic fecal DNA contains higher counts of CpG, compared to non-diabetic fecal DNA, and that BMDMs stimulated with diabetic fecal DNA present a higher production of TLR9-dependent type I interferon, which augments AIM2 expression and leads to a greater IL-1β and IL-18 production. However, our results show that only IL-1β production depends on AIM2 activation, since AIM2^−/−^ BMDMs, after several stimuli, produced lower levels of IL-1β, compared to the BMDMs from WT mice. There are two possibilities to explain why IL-18 production in vitro induced by diabetic fecal DNA depends on AIM2. One is that other receptors are involved in IL-18 production in vitro, and the other is that the assay used to determine the IL-18 levels does not discriminate between the inactive and active forms of IL-18, with the latter being a marker for AIM2 inflammasome activation.

In summary, our results demonstrate that the activation of AIM2 in the small intestine leads to IL-18 release, which regulates the expression of ZO-1, claudin-2 and RegIIIγ expression, thus improving the intestinal barrier function, which controls gut microbiota translocation to PLNs and the generation of a proinflammatory response against insulin-producing β cells, thus delaying STZ-induced T1D progression in our murine model. Overall, our study provides a new mechanism by which the innate immune receptor, AIM2, protects against STZ induced-T1D by regulating intestinal homeostasis and inhibiting gut microbiota translocation, events that are strongly associated with the development of autoimmune disorders.

## List of Abbreviations

Abx	Antibiotics
APC	Antigen-presenting cells
AIM2	Absent in melanoma 2
BMDM	Bone Marrow-Derived Macrophages
CAD	Diabetic ketoacidosis
CARD	Domain of the recruitment and activation of caspase
CD	Cluster of differentiation
NK Cells	Natural Killer Cells
DC	Dendritic cell
T1DM	Type 1 Diabetes Mellitus
DNA	Deoxyribonucleic acid
SD	Standard deviation E
FIIND	Domain function to find G
GAD	Glutamic acid decarboxylase
HLA	Human leukocyte antigen
IFN-γ	Interferon gamma
IL	Interleukin
PLNs	Pancreatic lymph nodes
LPS	Lipopolysaccharide
LRR	Leucine-rich repeats
MHC-II	Major Histocompatibility Complex-II
NF-κB	Nuclear Factor κB
NLR	NOD-like Receptor
NLRP1	NLR family pyrin domain containing 1
NOD	Non-obese diabetic mouse
NOR	Non-obese resistant mouse
PAMPs	Molecular patterns associated with the pathogen
PBMC	Peripheral blood mononuclear cells
PCR	Polymerase Chain Reaction
PRR	Pattern Recognition Receivers
PYD	Domain pyrin
RNA	Ribonucleic acid
sgRNA	Short-guide RNA
SNP	Single-nucleotide polymorphism
STZ	Streptozotocin
Th	T cell helper
TLR	Toll-like Receptor
TNF-α	Tumor necrosis factor alpha
WT	Wild type
